# Effects of Lactobacillus crispatus–Containing Probiotics and Metformin on Insulin Resistance, Inflammation, and Gut Microbiome in Polycystic Ovary Syndrome: Protocol for an Open-Label Randomized Controlled Trial

**DOI:** 10.2196/102121

**Published:** 2026-11-05

**Authors:** Bhuvaneswari H, Suchanda Sahu, Deepthy Balakrishnan, Balamurugan Ramadass

**Affiliations:** 1 Department of Biochemistry, All India Institute of Medical Sciences Bhubaneswar, Academic Block, Sijua, PO Dumduma, Bhubaneswar, Odisha, 751019, India, 91 9438884031; 2 Department of Obstretics and Gynaecology, All India Institute of Medical Sciences Bhubaneswar, Bhubaneswar, Odisha, India

**Keywords:** polycystic ovary syndrome, PCOS, gut microbiome, metformin, insulin resistance, acylated ghrelin, probiotic therapy, inflammation, polyendocrine metabolic ovarian syndrome, PMOS

## Abstract

**Background:**

Polycystic ovary syndrome (PCOS) affects 9% to 12% of reproductive-age women worldwide, with higher prevalence reported in India. Its pathophysiology involves insulin resistance, hyperandrogenism, and chronic low-grade inflammation, increasingly linked to gut dysbiosis and lipopolysaccharide-driven toll-like receptor 4 signaling. Elevated calprotectin and suppressed acylated ghrelin (AGH) reflect this inflammatory-metabolic disruption. While metformin remains standard therapy, it does not address underlying gut dysbiosis. *Lactobacillus crispatus*, with its antibacterial and immunomodulatory properties, is a promising but untested gut-targeted adjunct. This trial evaluates whether combining metformin with *L. crispatus* supplementation improves metabolic and inflammatory outcomes beyond either therapy alone.

**Objective:**

This study aims to compare probiotic, metformin, and combination therapy for reducing insulin resistance in PCOS over 12 weeks and assess their effects on gut barrier integrity, microbiome composition, hyperandrogenemia, glycemic control, and clinical outcomes.

**Methods:**

This open-label randomized, hospital-based trial at the All India Institute of Medical Sciences, Bhubaneswar, will assess the effects of probiotic and/or metformin therapy in women aged 18 to 40 years diagnosed with PCOS per Rotterdam criteria. A total of 135 participants will be randomized equally into 3 groups: probiotics only, metformin only, and combination therapy. The 3-month intervention includes metformin (1000 mg per day) and a specified *L. crispatus*–containing probiotic (2 capsules per day). The primary outcome is the change in Homeostatic Model Assessment for Insulin Resistance (HOMA-IR). Key secondary outcomes include changes in serum and fecal calprotectin, AGH, total testosterone, and fecal microbiome profile.

**Results:**

Participant recruitment began in January 2026. As of August 30, 2026, 26 of the planned 135 participants had been enrolled. Enrollment and data collection are projected to conclude by December 2026; statistical analysis will begin after database lock, with study completion anticipated by June 2027 and publication targeted for mid- to late 2027.

**Conclusions:**

This trial will provide critical, mechanistic evidence on whether the targeted action of *L. crispatus* can work additively with metformin to heal the intestinal mucosal barrier, arrest systemic endotoxemia, and rescue disrupted gut-brain-endocrine signaling. By validating this novel metabolic-inflammatory regimen against localized (calprotectin) and systemic (AGH and HOMA-IR) end points, this study intends to establish a highly integrated, microbiome-targeted therapeutic strategy capable of optimizing long-term metabolic and reproductive health outcomes in women with PCOS.

## Introduction

### Background

Polycystic ovary syndrome (PCOS) is the most common endocrine and metabolic disorder affecting women of reproductive age, with a pooled global prevalence estimated at 9% to 12% using the Rotterdam criteria [1,2]. In India specifically, hospital-based and community cohorts report a comparable or higher burden, with pooled estimates ranging from 11% to nearly 20% depending on the population studied and the diagnostic criteria applied [3,4]. Clinically, PCOS manifests with a spectrum of symptoms, including irregular menstruation, hirsutism, infertility, acne, and obesity. Diagnosis is established using the Rotterdam criteria, which require the presence of at least two of the following: (1) oligo- or anovulation (cycles of >35 days apart or <8 menses a year), (2) clinical or biochemical hyperandrogenism, and (3) polycystic ovarian morphology (≥20 follicles per ovary in either ovary and ≥10-cm^3^ ovarian volume) [5,6]. Women with PCOS are 2 to 6 times more likely to develop type 2 diabetes, hypertension, dyslipidemia, endometrial cancer, and cardiovascular diseases compared to the general population [7].

The etiology of PCOS is multifactorial, involving genetic predisposition and environmental influences. Its pathophysiology is characterized by hormonal imbalance, chronic low-grade inflammation, insulin resistance, and hyperandrogenism, which collectively disrupt normal folliculogenesis and increase the risk of long-term comorbidities. Recent studies underscore the critical role of gut microbiota in metabolic regulation. An imbalance in gut microbial composition termed gut dysbiosis is increasingly recognized as a contributing factor in PCOS pathogenesis. Dysbiosis, marked by a reduced *Bacteroidetes*-to-*Firmicutes* ratio, depletion of *Akkermansia muciniphila*, and an overgrowth of proteobacteria, compromises intestinal barrier integrity and facilitates the translocation of endotoxins such as lipopolysaccharides (LPSs) into systemic circulation [8-10].

According to the dysbiosis of gut microbiota hypothesis, this LPS translocation activates toll-like receptor 4 (TLR4) signaling, driving systemic low-grade inflammation, exacerbating insulin resistance, and stimulating ovarian androgen synthesis [9,11]. Local toll-like receptor 2 (TLR2) and TLR4 activation at the gut mucosa triggers nuclear factor kappa-light-chain-enhancer of activated B cells (NF-κB)–dependent proinflammatory cytokines (including interleukin-8 [IL-8]) that further disrupt epithelial tight junctions, creating a self-perpetuating cycle of barrier dysfunction. In this context, calprotectin—a calcium-binding protein released by neutrophils and monocytes during inflammatory cascades [12,13]—serves as a sensitive biomarker for mucosal and systemic inflammation. Elevated serum and fecal calprotectin levels in women with PCOS directly mirror this underlying inflammatory activity and intestinal permeability [14,15]. Concurrently, metabolic signaling along the gut-brain-endocrine axis is severely disrupted in PCOS, particularly regarding ghrelin dynamics. Acylated ghrelin (AGH), the biologically active form of ghrelin, is essential for regulating appetite, energy expenditure, and glucose homeostasis. Beyond its metabolic functions, AGH exerts potent anti-inflammatory effects by binding to growth hormone secretagogue receptors on immune cells, thereby deactivating the NF-κB pathway and polarizing macrophages toward a protective M2 phenotype [16]. However, the persistent hyperinsulinemia characteristic of PCOS chronically suppresses circulating AGH levels, depriving the host of a critical metabolic and anti-inflammatory checkpoint. Consequently, a suppressed AGH profile reflects advanced insulin resistance and unmitigated chronic inflammation in this population [16,17]. Metformin, a biguanide insulin-sensitizing agent, remains the cornerstone of pharmacological management in PCOS. By activating adenosine monophosphate–activated protein kinase, metformin reduces hepatic gluconeogenesis and enhances peripheral glucose uptake [18,19]. The resulting drop in circulating insulin suppresses ovarian androgen synthesis, restoring ovulatory function and menstrual regularity [20,21]. Despite its established metabolic efficacy, metformin monotherapy leaves upstream gut dysbiosis and intestinal barrier breakdown largely unmanaged [22]. Probiotic supplementation has emerged as a promising strategy to modulate the gut-metabolic axis. Multiple randomized controlled trials (RCTs) demonstrate that probiotics, typically containing *Lactobacillus* and *Bifidobacterium* species, improve insulin sensitivity, androgen profiles, and systemic inflammatory markers in women with PCOS [23,24]. Proposed mechanisms include competitive exclusion of pathobionts, tight junction reinforcement, enhanced mucin production, and modulation of short-chain fatty acid availability [25]. Among potential candidates, *Lactobacillus crispatus*, a dominant commensal species, possesses unique immunomodulatory properties. Its surface-layer proteins shield its own TLR2 ligands while selectively engaging the anti-inflammatory receptor dendritic cell-specific intercellular adhesion molecule-3-grabbing nonintegrin, preventing NF-κB activation and lowering proinflammatory cytokines such as interleukin-1 beta and IL-8 [26]. Furthermore, *L. crispatus* exhibits direct antibacterial activity against LPS-producing Gram-negative organisms (eg, *Escherichia coli*) via specialized bacteriocin gene clusters [27]. Importantly, *L. crispatus* is not restricted to the reproductive tract; gastrointestinal isolates share high genomic homology with vaginal strains, survive gastric acid and bile exposure, and improve insulin sensitivity and hepatic steatosis in preclinical models [27].

Despite these mechanistic advantages, *L. crispatus* has not yet been evaluated as a gut-targeted intervention in PCOS. Combining metformin with *L. crispatus* formulation represents a novel therapeutic approach to simultaneously address systemic glycemic dysregulation and upstream gastrointestinal inflammation. By tracking fecal and serum calprotectin alongside the AGH axis, this trial aims to evaluate whether a synchronized metabolic-inflammatory regimen offers additive clinical benefits over standard monotherapy.

To test this hypothesis, the present protocol details the PCOME (PCOS Combination (Probiotic+Metformin) Outcome in Metabolic and Endocrine) study**,** a randomized, open-label clinical trial designed to evaluate the individual and combined effects of *L. crispatus*–containing probiotic supplementation and metformin therapy. By integrating detailed metabolic, hormonal, and microbiome profiling end points, the PCOME study aims to advance a more precise, holistic framework for the management of PCOS.

### Study Objectives

#### Primary Objective

The primary objective is to compare the therapeutic efficacy of probiotic monotherapy, metformin monotherapy, and combination therapy in reducing insulin resistance in women with PCOS over a 3-month intervention period.

#### Secondary Objectives

Secondary objectives are as follows:

1. Gut-immune axis modulation: to evaluate whether probiotic supplementation (alone or adjunctive to metformin) attenuates gut mucosal inflammation and restores intestinal barrier integrity
2. Microbiome remodeling: to determine the effect of the 3 intervention regimens on gut microbial diversity, structural composition, and short-chain fatty acid metabolic output
3. Endocrine and metabolic regulation: to compare the impact of single and combined therapies on systemic hyperandrogenemia and secondary glycemic control
4. Clinical efficacy: to evaluate the comparative effectiveness of the 3 regimens in improving clinical features of PCOS, specifically cutaneous hyperandrogenism and menstrual cycle regularity

## Methods

### Study Design and Setting

The PCOME study is a 3-month, hospital-based open-label randomized clinical trial. This single-center study is being conducted at the All India Institute of Medical Science (AIIMS), Bhubaneswar, India. Participant recruitment will take place at the Department of Obstetrics and Gynaecology, AIIMS, Bhubaneswar. Sample collection, processing, and laboratory analyses will be performed at the Department of Biochemistry, the Central Research Laboratory, and the Centre of Excellence for Clinical Microbiome Research of the AIIMS, Bhubaneswar.

### Ethical Considerations

The study protocol has been reviewed and approved by the Institutional Ethics Committee of the AIIMS, Bhubaneswar, under reference number IEC/AIIMS BBSR/PG Thesis/2025-26/04, dated August 4, 2025. All eligible participants will receive detailed information regarding the study objectives, procedures, and potential risks. Written informed consent will be obtained before enrollment and randomization. The trial has been prospectively registered with the Clinical Trials Registry - India under the registration number CTRI/2025/09/094113. Patients, Exeltis India, or the public were not involved in our research’s design and will not be involved in conduct, reporting, or dissemination plans.

### Participants

#### Inclusion Criteria

Participants are eligible for inclusion if they meet all the following criteria:

- Female patients aged 18 to 40 years
- Diagnosis of PCOS based on the Rotterdam criteria (requiring the presence of at least 2 of the following: oligo- or anovulation, clinical or biochemical hyperandrogenism, and polycystic ovarian morphology)
- Willingness to participate and provide written informed consent

#### Exclusion Criteria

Participants are excluded if they meet any of the following criteria:

- Presence of comorbid conditions that may confound PCOS diagnosis or metabolic outcomes, including diabetes mellitus, hypo- or hyperthyroidism, Cushing syndrome, or other causes of secondary hyperandrogenism
- Current pregnancy or lactation
- Use of medications or supplements that may alter gut microbiota or hormonal or metabolic parameters
- Use of antibiotics, probiotics, or prebiotics within 3 months prior to screening
- Use of oral proton-pump inhibitors or laxatives within 3 months prior to screening
- Use of drugs for the treatment of acne, alopecia, or hirsutism (eg, antiandrogens) within 6 months prior to screening
- Hormonal treatments (eg, oral contraceptive pills) initiated before or during the study period
- Known allergy or intolerance to metformin or any component of the probiotic formulation

### Sample Size Calculation

The sample size for the PCOME study was estimated based on the primary objective: to detect a statistically significant difference in the change in Homeostatic Model Assessment for Insulin Resistance (HOMA-IR) at 3 months, specifically comparing the combination therapy group (probiotic+metformin) to the metformin-only group.

Because the primary analysis is a baseline-adjusted analysis of covariance (ANCOVA; 12-week HOMA-IR as the dependent variable, treatment group as the fixed factor, and baseline HOMA-IR as a covariate), the sample size was calculated using the corresponding ANCOVA design effect formula, which accounts for the correlation between baseline and outcome measurements:


$$n=\frac{2{\left(Z_{1-\alpha /2}+Z_{1-\beta }\right)}^{2}\sigma^{2}(1-\rho^{2})}{\delta^{2}}$$

The primary end point is the change in HOMA-IR from baseline to 3 months. Significance level (Z_1 – α_/2) was set at 1.96 (2-tailed α=.05). Power (Z_1 – β_) was set at 0.84 (80% power). The SD (σ) was set at 1.5 units.

The minimal clinically important difference (δ) is 0.65 units between the combination and metformin-only groups. This value falls within the 95% CI (−0.73 to −0.26) of the pooled HOMA-IR mean difference (−0.50) reported in a 4-study meta-analysis (n=527) comparing probiotic plus metformin combination therapy to metformin alone.

The baseline-to-outcome correlation (ρ) is 0.75, assumed based on the general short-interval stability of insulin resistance markers. This value is a working assumption pending confirmation from the trial’s own paired baseline and 12-week data (see the Sensitivity Analysis of ρ section).

The unadjusted sample size per group was calculated as follows: n = 2(1.96 + 0.84)^2^ × 1.5^2^ × (1 − 0.75^2^) / 0.65^2^ ≈ 37. After adjustment for a 16% anticipated dropout, the sample size was as follows: n = 37/(1 − 0.16) ≈ 45 per group.

The final enrollment target remains 45 participants per group. A total of 135 participants will be recruited and randomized equally across the 3 intervention arms: probiotic only (group A), metformin only (group B), and combination therapy (probiotic+metformin; group C).

### Study Design and Products

The PCOME study is a 12-week (3-month), single-center open-label RCT. The 3 intervention groups will receive probiotic capsules (2 capsules per day; group A), 500 mg of metformin twice daily (group B), or probiotic capsules (2 capsules per day) plus 500 mg of metformin twice daily (group C). All enrolled participants are being followed for the full 3-month intervention period. The study flowchart is shown in Figure 1.

**Figure 1.**
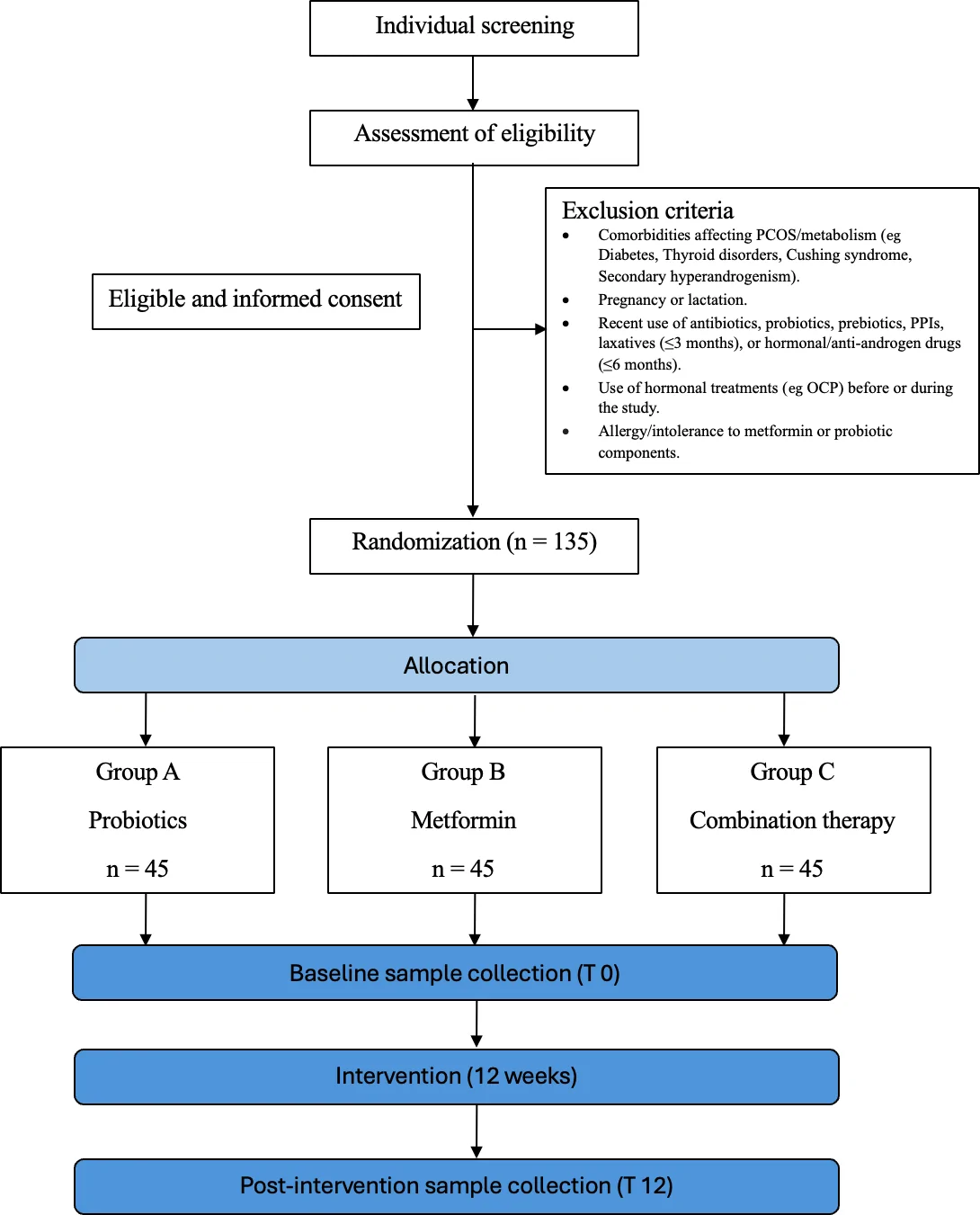
Study flowchart: screening, randomization, and intervention allocation. This flowchart illustrates the sequential steps of the PCOME study, beginning with individual screening and eligibility assessment in the Obstetrics and Gynaecology Outpatient Department. Participants who meet the inclusion criteria and provide informed consent are randomized (N=135) into 3 intervention arms: group A (probiotics), group B (metformin), and group C (combination therapy). Baseline sample collection (T0) is followed by a 12-week intervention period, culminating in postintervention sample collection (T12). The diagram also outlines exclusion criteria, including metabolic comorbidities, recent antibiotic or probiotic use, and contraindications to study products. This structured design ensures rigorous participant selection and standardized data collection across all groups. OCP: oral contraceptive pill; PCOS: polycystic ovary syndrome; PPI: proton-pump inhibitor.

### Treatment Allocation and Randomization

Eligible patients who provide informed consent will be assigned a unique study code. Participants will be randomly assigned to group A, group B, or group C in equal numbers (1:1:1 ratio), targeting 45 participants per group (N=135). Before the trial began, the randomization list was created using software (Microsoft Excel) with block randomization (block size of 12) to ensure balanced groups throughout recruitment. To keep assignments hidden until enrollment, we will use sequentially numbered, opaque, sealed envelopes. Each envelope will contain the group assignment and be numbered from 1 to 135. Envelopes will only be opened after a participant meets all study criteria, signs the consent form, and is enrolled. Recruiting researchers will not have access to the master list.

### Intervention Details

#### Probiotic Monotherapy (Group A)

Participants will receive a probiotic capsule (V-pH, manufactured by Ordain Health Care Global Pvt Ltd/Exeltis). Each capsule contains probiotic formulation totaling 2.5 billion colony-forming units (CFU), comprising 1 billion CFU of *L. crispatus*, 1 billion CFU of *Lactobacillus rhamnosus*, 0.3 billion CFU of *Lactobacillus gasseri*, and 0.2 billion CFU of *Lactobacillus jensenii*. Participants will take 2 capsules daily (receiving a total dose of 5 billion CFU per day) for 12 weeks.

#### Metformin Monotherapy (Group B)

Participants will receive 500-mg tablets of generic metformin hydrochloride procured via the institutional central pharmacy (AIIMS, Bhubaneswar). Participants will take 1 tablet twice daily after meals (1000 mg per day in total) for 12 weeks.

#### Combination Therapy (Group C)

Participants will receive both the probiotic regimen (V-pH; 5 billion CFU per day) and metformin (1000 mg per day) concurrently for 12 weeks.

#### Dose Justification

The formulation delivers *L. crispatus* at 1 × 10^9^ CFU per capsule and 2 capsules daily. This provides 2 × 10^9^ CFU per day. No prior trial has evaluated *L. crispatus* specifically in PCOS, so this dose was based on the established per-strain effective dose for other *Lactobacillus* and *Bifidobacterium* species in PCOS RCTs. Karamali et al [23] and Ostadmohammadi et al [24] each dosed their constituent strains at 2 × 10^9^ CFU per gram over 12 weeks, achieving significant improvements in testosterone, sex hormone–binding globulin (SHBG), high-sensitivity C-reactive protein, and oxidative stress markers vs placebo. As *L. crispatus* is delivered in the same genus and formulation class, 2 × 10^9^ CFU per day was selected as a dose with established precedent for metabolic and hormonal benefit in PCOS at the genus level.

Metformin was dosed at 500 mg twice daily (1000 mg per day), consistent with the work by Kuang et al [28], in which this dose combined with probiotic supplementation produced significant improvements in fasting glucose, 2-hour postprandial glucose, and HOMA-IR by 1 month, with sustained metabolic and hormonal benefits up to 12 weeks. This dose is also supported by a 24-week phase 3 RCT in which the same regimen (500 mg of metformin twice daily) produced HOMA-IR improvement in 56.18% of patients by 12 weeks, rising to 60.67% by 24 weeks, alongside improved menstrual cycle regularity [29]. The dose of 1000 mg per day was selected to balance efficacy with tolerability: gastrointestinal adverse effects associated with metformin are dose dependent, and a recent meta-analysis reported a 7.7-fold increased risk of mild gastrointestinal effects with metformin vs placebo [30]. Selecting a moderate, well-established dose was therefore prioritized to support adherence and minimize the risk of treatment discontinuation over the study period.

### Intervention Initiation and Monitoring

Supplementation will begin immediately after formal PCOS diagnosis and randomization. Clinical supplies will be dispensed monthly for the 3-month duration. Treatment adherence will be monitored through monthly hospital visits and reinforced via regular phone calls by study investigators.

### Data Collection and Management

The timeline of follow-up visits and sample collection is illustrated in Figure 2. The schedule of assessments and measurements is detailed using the SPIRIT (Standard Protocol Items: Recommendations for Interventional Trials) guidelines shown in Table 1, and secondary outcome measures are shown in Table 2.

**Figure 2.**
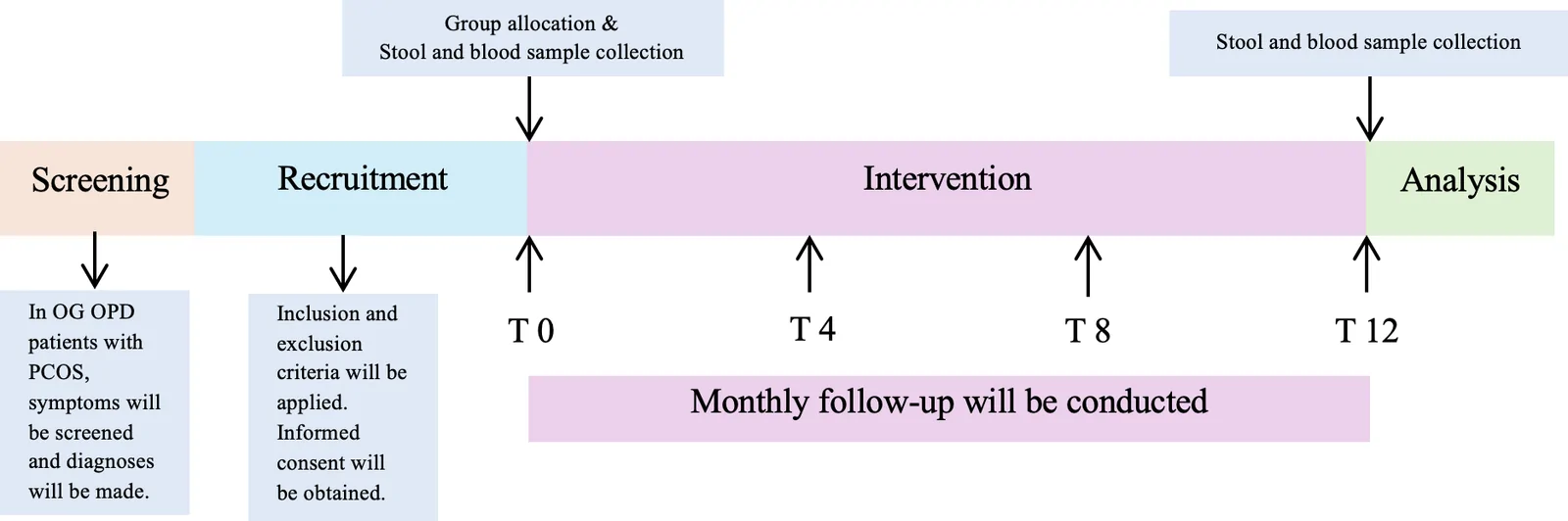
Schematic representation of study design and timeline. This flowchart outlines the sequential phases of the PCOME (PCOS Combination (Probiotic+Metformin) Outcome in Metabolic and Endocrine) study: screening, recruitment, intervention, and analysis. Patients presenting to the Obstetrics and Gynaecology Outpatient Department (OG OPD) are screened and diagnosed with polycystic ovary syndrome (PCOS). Eligible participants are recruited based on the inclusion and exclusion criteria, followed by randomization and baseline sample collection (T0). The intervention phase includes monthly follow-up visits at T4 and T8. Final assessments and sample collection are conducted at T12. Blood and stool samples are collected at T0 and T12 for biochemical and microbiome analyses. This timeline ensures structured data collection to evaluate the metabolic, hormonal, and inflammatory effects of the interventions over the 12-week period.

**Table 1.** Schedule of enrollment, interventions, and assessments^a^.

	Enrollment (–T1)	Allocation	After allocation		
		T0	T4	T8	T12
Enrollment					
Eligibility screen	✓				
Informed consent	✓				
Randomization	✓				
Allocation		✓			
Intervention					
Metformin		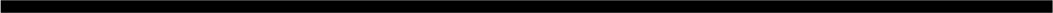			
Probiotics		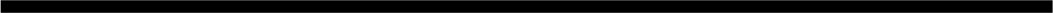			
Metformin+probiotics		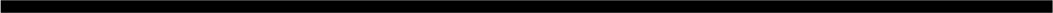			
Assessments					
Fecal calprotectin		✓			✓
Acylated ghrelin		✓			✓
Insulin resistance markers		✓			✓
Hyperandrogenism markers		✓			✓

a This table outlines the study timeline across 5 key time points (−T1, T0, T4, T8, and T12), corresponding to the enrollment, allocation, and postallocation phases of the 12-week PCOME trial. Activities include eligibility screening, informed consent, randomization, and intervention initiation. The intervention arms—metformin, probiotics, and combination therapy—are administered after allocation. Scheduled assessments include fecal calprotectin, serum acylated ghrelin, insulin resistance markers (eg, fasting glucose, insulin, and Homeostatic Model Assessment for Insulin Resistance), and hyperandrogenism markers (eg, testosterone and luteinizing hormone–to–follicle-stimulating hormone ratio) performed at designated time points to evaluate therapeutic impact.

**Table 2.** Secondary outcome measures assessed from baseline to 12 weeks^a^.

Category	Specific outcome measures (change from baseline to 12 weeks)
Inflammatory and gut health markers	Serum calprotectin Fecal calprotectin Serum AGH^b^ Fecal SCFA^c^ concentrations Serum zonulin levels (marker of intestinal permeability)
Metabolic and IR^d^ markers	Fasting glucose Fasting insulin HbA_1c_^e^
Hyperandrogenism and hormonal markers	LH^f^ FSH^g^ LH-to-FSH ratio SHBG^h^ Total testosterone
Gut microbiome profile	Fecal microbiome alpha and beta diversity Differential abundance of key bacterial taxa (eg, *Bacteroidetes*-to-*Firmicutes* ratio and *Lactobacillus* species)
Clinical outcomes	BMI and WHR^i^ Modified Ferriman-Gallwey score (hirsutism assessment) Menstrual regularity

a This table categorizes the secondary outcomes evaluated in the PCOME study, reflecting the multidimensional impact of probiotic and/or metformin therapy on women with polycystic ovary syndrome. Outcomes are grouped into 5 domains: inflammatory and gut health markers, metabolic and insulin resistance indicators, hormonal and hyperandrogenism profiles, gut microbiome characteristics, and clinical end points. Each parameter will be measured at baseline and after the 12-week intervention period to assess therapeutic efficacy and mechanistic modulation.

b AGH: acylated ghrelin.

c SCFA: short-chain fatty acid.

d IR: insulin resistance.

e HbA_1c_: hemoglobin A_1c_.

f LH: luteinizing hormone.

g FSH: follicle-stimulating hormone.

h SHBG: sex hormone–binding globulin.

i WHR: waist-to-hip ratio.

### Compliance and Analysis Set

Compliance with the study interventions (metformin and/or probiotics) will be rigorously monitored through monthly follow-up visits and regular phone calls. Participants with less than 80% adherence will be documented and may be excluded from the final primary outcome analysis. Results will be analyzed using both per-protocol and intention-to-treat (ITT) approaches, as recommended for nutrition and mechanistic trials.

### Safety Monitoring and Adverse Events

Participants will be proactively instructed to report any unexpected symptoms or discomfort associated with the study interventions and will be counseled regarding common, transient gastrointestinal effects that may occur during the initial phase of probiotic or metformin supplementation, such as mild flatulence or bloating. Safety and drug tolerability are formally assessed at weeks 4, 8, and 12, with participants recording any symptoms (eg, nausea, bloating, or diarrhea) using standard daily logs between visits. During each follow-up visit, study investigators will actively inquire about any adverse events (AEs) potentially related to the intervention. For every reported AE, investigators will document its severity, duration, treatment required, and assessed causality; all AEs will be recorded in detail and included in the final study report. Any severe AEs will be reported immediately to the Institutional Ethics Committee of the AIIMS, Bhubaneswar. The likelihood of serious AEs is considered low given that both the metformin dose and the probiotic strains used are well established, commercially available, and generally recognized as safe.

### Sample Collection and Laboratory Analysis

Stool samples will be collected at 2 time points: baseline (T0; after PCOS diagnosis but before intervention initiation) and after the intervention (T12; at the end of the 12-week supplementation period). Participants will receive sterile stool collection kits and be instructed to store samples in sealed containers in home freezers (≤−20 °C). Samples will be transported to the laboratory in insulated carriers and stored at −80 °C until analysis. Initial analysis will focus on fecal calprotectin using a validated enzyme-linked immunosorbent assay.

### Gut Health Survey

#### Overview

The modified Gastrointestinal Symptoms Rating Scale (mGSRS) will be administered at T0 and T12 to assess gut health. The mGSRS includes questions on general gastrointestinal symptoms, stool frequency and consistency (via the Bristol stool scale), and perceived bowel habit normality (Likert scale). Details are provided in Multimedia Appendix 1.

#### Blood Collection and Analysis

Venous blood (10 mL) will be collected at T0 (baseline) and T12 (end of study). Blood will be drawn into clot activator tubes (for serum), sodium fluoride tubes (for plasma glucose), and ethylenediaminetetraacetic acid tubes (for plasma, if required). Samples will be centrifuged within 2 hours, aliquoted into Eppendorf tubes, and snap frozen at −80 °C until analysis. The plasma and serum specimens will be analyzed as shown in Table 3.

**Table 3.** Analytical methods and instrumentation for biochemical assays^a^.

Parameter	Assay method	Analyzer
Fasting glucose	GOD-POD^b^ method	Beckman Coulter AU5800 automatic analyzer
HbA_1c_^c^	IFCC^d^ turbidimetry immunoinhibition method	Beckman Coulter AU5800 automatic analyzer
Fasting insulin, total testosterone, LH^e^, and FSH^f^	ECLIA^g^	Cobas e 801 automatic analyzer
Fecal calprotectin	Validated ELISA^h^	—^i^
AGH^j^	Validated ELISA	—
Serum zonulin levels	Validated ELISA	—
SHBG^k^	Validated ELISA	—
Fecal SCFAs^l^	—	—
Gut microbiome profile	16S rRNA^m^-based amplicon sequencing	Illumina NexSeq 2000

a This table summarizes the assay techniques and analyzers used for quantifying key biochemical parameters in the PCOME study.

b GOD-POD: glucose oxidase-peroxidase.

c HbA_1c_: hemoglobin A_1c_.

d IFCC: International Federation of Clinical Chemistry and Laboratory Medicine.

e LH: luteinizing hormone.

f FSH: follicle-stimulating hormone.

g ECLIA: electrochemiluminescence immunoassay.

h ELISA: enzyme-linked immunosorbent assay.

i Not applicable.

j AGH: acylated ghrelin.

k SHBG: sex hormone–binding globulin.

l SCFA: short-chain fatty acid.

m rRNA: ribosomal RNA.

#### Calculated Parameters

The calculated parameters are as follows:

- HOMA-IR: the HOMA-IR will be calculated using fasting glucose and fasting insulin values applying the standard formula of HOMA-IR = fasting insulin (µU/mL) × fasting glucose (mg/dL)/405.
- Luteinizing hormone (LH)–to–follicle-stimulating hormone (FSH) ratio: the LH-to-FSH ratio will be derived from the measured serum concentrations of LH and FSH, expressed as a unitless ratio.
- Free androgen index: this will be calculated as (total testosterone/SHBG) × 100.

### Clinical Data

Patient history, anthropometric measurements (BMI and waist-to-hip ratio), and biochemical data will be recorded in a dedicated case report form.

### Statistical Analysis

#### Overview

As mentioned, sample size was calculated using the ANCOVA design effect formula n = 2(Z_1 – α_/2 + Z_1 – β_)^2^ × σ^2^ × (1 − ρ^2^)/δ^2^, reflecting the trial’s planned baseline-adjusted primary analysis (12-week HOMA-IR as the dependent variable, treatment group as the fixed factor, and baseline HOMA-IR as the covariate) rather than a plain 2–independent means comparison. Using a 2-tailed α of .05, power of 80%, σ of 1.5 units, δ of 0.65 units, and an assumed baseline-to-outcome correlation of ρ=0.75, a minimum of 111 participants (n=37 per group) was estimated to achieve 80% power at a 95% confidence level for the primary HOMA-IR comparison. Accounting for a 16% anticipated dropout rate, the final sample size was set at 135 participants (n=45 per group). Data will be analyzed using SPSS Statistics (version 26.0; IBM Corp) or an equivalent statistical software. All entries will be maintained in a secure Microsoft Excel database before analysis.

#### Primary Estimand Framework

The primary estimand is the between-group difference in mean change in HOMA-IR from baseline to 12 weeks comparing the combination therapy group to the metformin monotherapy group. The primary analysis will be conducted under the ITT principle, with a per-protocol analysis performed as a sensitivity check.

As a parallel 3-arm trial without a placebo arm, this design evaluates comparative clinical effectiveness across the 3 regimens; it is not powered or structured to statistically break down a true pharmacological interaction (synergy) between probiotics and metformin. Findings of statistically superior improvement in the combination group relative to either monotherapy will therefore be interpreted as evidence of additive clinical benefit, not confirmed synergy.

The combination vs metformin contrast is designated as the primary comparative hypothesis, directly reflecting the sample size calculation above. Secondary pairwise comparisons (combination vs probiotic monotherapy and probiotic monotherapy vs metformin monotherapy) will be interpreted using a Bonferroni-adjusted significance threshold.

#### Sensitivity Analysis of ρ

As sufficient paired baseline and 12-week HOMA-IR data accumulate from enrolled participants, the observed baseline-to-outcome correlation (ρ) will be calculated directly from trial data and compared against the assumed value (ρ=0.75) used in the sample size calculation. This will be reported as a prespecified sensitivity analysis. If the observed ρ is materially lower than assumed, achieved statistical power for the primary comparison will be recalculated using the observed value and reported alongside the primary result.

#### Analysis Populations and Missing Data

The ITT population will comprise all randomized participants who receive at least one dose and have baseline data.

The per-protocol population will comprise randomized participants who complete the 12-week protocol without major deviations and achieve 80% or higher intervention adherence (verified via pill or sachet counts).

#### Descriptive Statistics

Normality will be evaluated using *Q*-*Q* plots and the Shapiro-Wilk test. Continuous variables will be summarized as means and SDs (normal distribution) or medians with IQRs (nonnormal distribution). Categorical variables will be summarized as frequencies and percentages.

#### Comparative and Multivariable Analyses

Baseline characteristics will be compared using one-way ANOVA, Kruskal-Wallis tests, or chi-square or Fisher exact tests, as appropriate.

#### Within-Group Changes: Paired 2-tailed *t* Tests or Wilcoxon Signed-Rank Tests

For between-group primary effects (ANCOVA), 12-week HOMA-IR will be the dependent variable, treatment group will be the fixed factor, and baseline HOMA-IR will be the covariate. Baseline-adjusted mean differences will be reported with 95% CIs. For nonnormal residuals, log transformation of HOMA-IR or a Kruskal-Wallis test with Dunn-Bonferroni post hoc comparisons will be applied.

#### Regression and Correlations

The Pearson *r* or Spearman ρ will be used, as appropriate; multivariate linear regression will be adjusted for baseline age, BMI, and other relevant covariates.

## Results

Participant recruitment officially initiated in January 2026. As of August 30, 2026, 26 participants of the target sample size of 135 had been successfully screened and enrolled. Data collection and follow-up assessments are being conducted per the protocol. No formal statistical or interim data analyses have been performed to date to maintain scientific integrity and prevent premature unblinding of trends. Sample collection and intervention phases are projected to conclude by December 2026. Formal data curation and statistical evaluations will commence immediately following final data lock, with overall study completion anticipated by June 2027. Full trial findings are targeted for publication in mid to late 2027.

## Discussion

### Study Rationale and Novelty

PCOS is a complex, multifactorial endocrine disorder characterized by a pathological interplay among chronic inflammation, insulin resistance, and hormonal dysregulation. These interwoven mechanisms disrupt ovarian function, metabolic homeostasis, and reproductive health. The PCOME study was conceived to evaluate therapeutic interventions within this integrative framework, recognizing that effective PCOS management must address not only clinical symptoms but also the underlying biochemical and microbial drivers of the disease. Emerging evidence underscores the pivotal role of gut dysbiosis in PCOS pathogenesis. Alterations in gut microbial composition can increase intestinal permeability, promote endotoxin translocation, and trigger systemic low-grade inflammation, factors that exacerbate insulin resistance and androgen excess. While standard clinical interventions rely on metformin to manage downstream glycemic and androgenic indexes, this monotherapy leaves the primary intestinal trigger completely unmanaged. Conversely, previous clinical trials evaluating probiotic supplementation have largely relied on broad-spectrum, generic formulas without quantifying their precise molecular impact. This study evaluates a targeted, strain-specific approach alongside highly localized and systemic biomarkers to demonstrate how repairing the intestinal architecture can restore systemic metabolic and reproductive homeostasis.

The PCOME study introduces a distinct therapeutic paradigm in PCOS management by shifting from empirical, broad-spectrum probiotic blends to a targeted, strain-specific formulation of *L. crispatus*. While conventional clinical approaches use metformin to address peripheral insulin sensitivity, they leave the upstream driver of gut dysbiosis and mucosal endotoxemia unmanaged. This robust, 3-arm RCT is uniquely positioned to evaluate the individual, additive effects of metformin and *L. crispatus* monotherapies against their concurrent coadministration over a 12-week period. The primary novelty of this framework lies in its multi-tiered, continuous evaluation of the gut-metabolic axis across 5 distinct biological dimensions. By integrating these precise end points, the trial tracks the resolution of localized intestinal wall injury alongside systemic immune stress by concurrently measuring fecal and serum calprotectin while simultaneously evaluating the restoration of the gut-brain-endocrine axis by monitoring the rescue of AGH levels. This inflammatory and endocrine mapping is coupled directly with the assessment of metabolic homeostasis via HOMA-IR, fasting insulin, and glucose dynamics alongside the normalization of ovarian steroidogenesis through total testosterone, SHBG, and the LH-to-FSH ratio. Finally, these systemic changes are correlated with structural shifts in microbial ecology by mapping the intestinal landscape through high-resolution microbiome profiling (alpha and beta diversity and key taxonomic abundance). Backed by a statistically rigorous design using HOMA-IR as a powered primary end point, the PCOME study provides the mechanistic depth required to determine whether a dual metabolic-inflammatory regimen can repair the intestinal architecture and restore ovarian steroidogenesis, ultimately redefining precision care for women with PCOS.

### Limitations

Despite its strengths, several limitations merit consideration. The sample size of 135 is adequately powered for the primary HOMA-IR outcome but may be underpowered for detecting subtle changes in secondary end points, particularly those with high biological variability, such as microbial taxa abundance.

An open-label design was selected due to the logistical infeasibility of manufacturing matching placebos that replicate the exact organoleptic properties (taste, appearance, and texture) of both active metformin and multi-strain probiotic interventions within an academic research framework.

To eliminate measurement bias, all laboratory and diagnostic analyses will be strictly blinded. Biological samples will be deidentified and assigned alphanumeric codes before processing. Laboratory personnel and assay operators evaluating biochemical markers, enzyme-linked immunosorbent assays, and microbiome profiles will remain completely blinded to group allocations throughout the study. The open-label design nonetheless introduces potential for participant or assessor bias in subjective measures (eg, hirsutism scores and mGSRS responses), although this is mitigated by the use of standardized biochemical and microbiome assays. Additionally, the 3-month follow-up period, while sufficient to capture early metabolic and inflammatory shifts, may not reflect long-term reproductive or cardiometabolic outcomes. Future longitudinal studies will be necessary to validate sustained clinical benefits and explore the durability of microbiome modulation.

### Conclusions and Future Directions

The PCOME study is positioned to make a meaningful contribution to the evolving landscape of PCOS research. By integrating gut health, metabolic regulation, and hormonal balance into a unified therapeutic model, it offers a blueprint for precision medicine in PCOS. Should combination therapy demonstrate superior efficacy, it will support the adoption of additive interventions that go beyond symptom management to address the root causes of the disease. The findings may inform clinical guidelines, inspire longer-term trials, and catalyze the development of microbiome-informed strategies for women’s endocrine health.

## Supplementary material

10.2196/102121 Multimedia Appendix 1 The Gastrointestinal Symptom Rating Scale (GSRS).

## Abbreviations

AE: adverse event
AGH: acylated ghrelin
AIIMS: All India Institute of Medical Science
ANCOVA: analysis of covariance
CFU: colony-forming unit
FSH: follicle-stimulating hormone
HOMA-IR: Homeostatic Model Assessment for Insulin Resistance
IL-8: interleukin-8
ITT: intention to treat
LH: luteinizing hormone
mGSRS: modified Gastrointestinal Symptoms Rating Scale
NF-κB: nuclear factor kappa-light-chain-enhancer of activated B cells
PCOME: PCOS Combination (Probiotic+Metformin) Outcome in Metabolic and Endocrine.
PCOS: polycystic ovary syndrome
RCT: randomized controlled trial
SHBG: sex hormone–binding globulin
SPIRIT: Standard Protocol Items: Recommendations for Interventional Trials
TLR2: toll-like receptor 2
TLR4: toll-like receptor 4
