# Supplementary material for: Effects of Lactobacillus crispatus–Containing Probiotics and Metformin on Insulin Resistance, Inflammation, and Gut Microbiome in Polycystic Ovary Syndrome: Protocol for an Open-Label Randomized Controlled Trial
Source: JMIR Res Protoc. 2026 Nov 5;15:e102121. doi: 10.2196/102121 (PMC13739260; doi:10.2196/102121)
Supplement: Supplementary file 1 [file resprot-v15-e102121-s001.docx]

**The Gastrointestinal Symptom Rating Scale (GSRS)**

GSRS score will be calculated as

Response scale

(1) No discomfort at all

(2) Slight discomfort

(3) Mild discomfort

(4) Moderate discomfort

(5) Moderately severe discomfort

(6) Severe discomfort

(7) Very severe discomfort

1. Have you been bothered by PAIN OR DISCOMFORT IN YOUR UPPER ABDOMENOR THE PIT

OF YOUR STOMACH during the past week?

2. Have you been bothered by HEARTBURN during the past week? (By heartburn we mean an unpleasant stinging or burning sensation in the chest.)

3. Have you been bothered by ACID REFLUX during the past week? (By acid reflux we mean the sensation of regurgitating small quantities of acid or flow of sour or bitter fluid from the stomach up

to the throat.)

4. Have you been bothered by HUNGER PAINS in the stomach during the past week? (This hollow feeling in the stomach is associated with the need to eat between meals.)

5. Have you been bothered by NAUSEA during the past week? (By nausea we mean a feeling of wanting to throw up or vomit.)

6. Have you been bothered by RUMBLING in your stomach during the past week? (Rumbling refers to vibrations or noise in the stomach.)

7. Has your stomach felt BLOATED during the past week? (Feeling bloated refers to swelling often associated with a sensation of gas or air in the stomach.)

8. Have you been bothered by BURPING during the past week? (Burping refers to bringing up air or gas from the stomach via the mouth, often associated with easing a bloated feeling.)

9. Have you been bothered by PASSING GAS OR FLATUS during the past week? (Passing gas or flatus refers to the need to release air or gas from the bowel, often associated with easing a bloated feeling.)

10. Have you been bothered by CONSTIPATION during the past week? (Constipation refers to a reduced ability to empty the bowels.)

11. Have you been bothered by DIARRHEA during the past week? (Diarrhea refers to a too frequent emptying of the bowels.)

12. Have you been bothered by LOOSE STOOLS during the past week? (If your stools (motions) have been alternately hard and loose, this question only refers to the extent you have been bothered by the stools being loose.)

13. Have you been bothered by HARD STOOLS during the past week? (If your stools (motions) have been alternately hard and loose, this question only refers to the extent you have been bothered by the stools being hard.)

14. Have you been bothered by and URGENT NEED TO HAVE A BOWEL MOVEMENT during the past week? (This urgent need to go to the toilet is often associated with a feeling that you are not in full control.)

15. When going to the toilet during the past week, have you had the SENSATION OF NOT

COMPLETELY EMPTYING THE BOWELS? (This feeling of incomplete emptying means that you still feel a need to pass more stool despite having exerted yourself to do so.)

**Calculation of GSRS-score**

Reflux-score: Average score of items 2 and 3

Abdominal pain-score: Average score of items 1, 4, and 5

Indigestion-score: Average score of items 6, 7, 8, and 9

Diarrhea-score: Average score of items 11, 12, and 14

Constipation-score: Average score of items 10,13, and 15

GSRS-score: Average of all five sub-scores

**Bristol stool Scale**

Type 1: Separate, hard lumps, like little pebbles

Type 2: Hard and lumpy, but sausage-shaped

Type 3: Sausage-shaped, with cracks on the surface

Type 4: Smooth, soft and snakelike

Type 5: Soft blobs with clear-cut edges

Type 6: Fluffy, mushy pieces with ragged edges

Type 7: Watery, liquid poop, with no solid pieces
